# Deep Learning-Based Computational Cytopathologic Diagnosis of Metastatic Breast Carcinoma in Pleural Fluid

**DOI:** 10.3390/cells12141847

**Published:** 2023-07-13

**Authors:** Hong Sik Park, Yosep Chong, Yujin Lee, Kwangil Yim, Kyung Jin Seo, Gisu Hwang, Dahyeon Kim, Gyungyub Gong, Nam Hoon Cho, Chong Woo Yoo, Hyun Joo Choi

**Affiliations:** 1 Department of Hospital Pathology, The Catholic University of Korea College of Medicine, Seoul 06591, Republic of Korea; griselbrand@gmail.com (H.S.P.); ychong@catholic.ac.kr (Y.C.); wondoocha@naver.com (Y.L.); kangse_manse@catholic.ac.kr (K.Y.); ywacko@catholic.ac.kr (K.J.S.); 2 AI Team, DeepNoid Inc., Seoul 08376, Republic of Korea; kisu031@gmail.com (G.H.); anniy8920@outlook.kr (D.K.); 3 Department of Pathology, Asan Medical Center, Seoul 05505, Republic of Korea; gygong@amc.seoul.kr; 4 Department of Pathology, Yonsei University College of Medicine, Seoul 03722, Republic of Korea; cho1988@yuhs.ac; 5 Department of Pathology, National Cancer Center, Ilsan, Goyang-si 10408, Gyeonggi-do, Republic of Korea; cwy@ncc.re.kr

**Keywords:** breast neoplasms, cytology, pleural fluid, artificial intelligence, metastasis, deep learning

## Abstract

A Pleural effusion cytology is vital for treating metastatic breast cancer; however, concerns have arisen regarding the low accuracy and inter-observer variability in cytologic diagnosis. Although artificial intelligence-based image analysis has shown promise in cytopathology research, its application in diagnosing breast cancer in pleural fluid remains unexplored. To overcome these limitations, we evaluate the diagnostic accuracy of an artificial intelligence-based model using a large collection of cytopathological slides, to detect the malignant pleural effusion cytology associated with breast cancer. This study includes a total of 569 cytological slides of malignant pleural effusion of metastatic breast cancer from various institutions. We extracted 34,221 augmented image patches from whole-slide images and trained and validated a deep convolutional neural network model (DCNN) (Inception-ResNet-V2) with the images. Using this model, we classified 845 randomly selected patches, which were reviewed by three pathologists to compare their accuracy. The DCNN model outperforms the pathologists by demonstrating higher accuracy, sensitivity, and specificity compared to the pathologists (81.1% vs. 68.7%, 95.0% vs. 72.5%, and 98.6% vs. 88.9%, respectively). The pathologists reviewed the discordant cases of DCNN. After re-examination, the average accuracy, sensitivity, and specificity of the pathologists improved to 87.9, 80.2, and 95.7%, respectively. This study shows that DCNN can accurately diagnose malignant pleural effusion cytology in breast cancer and has the potential to support pathologists.

## 1. Introduction

In 2020, breast cancer was the leading cancer worldwide and the fifth leading cause of cancer mortality [1]. Most of these deaths have been attributed to metastatic breast cancer. Breast cancer can spread to various organs in the body, with the most common sites being the bone, lung, liver, and brain [2,3]. In the United States, women with metastatic breast cancer have a 5-year survival rate of 30% [4]. The presence of malignant pleural effusions suggests advanced disease and is associated with poor survival rates [5]. Pleural metastasis occurs when cancer cells from a primary breast tumor spread to the pleural lining surrounding the lungs. Patients with breast cancer often experience pleural metastasis in the first few years after diagnosis, although this becomes less frequent with longer periods of disease-free survival [6]. Aspiration cytology is a widely used and efficient method for diagnosing malignant pleural effusions. This technique is recognized for its simplicity, cost-effectiveness, and rapid results [7]. Malignant pleural effusion cytology plays a pivotal role in the diagnosis, treatment planning, and monitoring of metastatic breast cancer. Patients with malignant effusions may also experience additional symptoms, such as dyspnea. Proper management of the respiratory decline can significantly enhance the patient’s quality of life. Timely information obtained from pleural effusion cytology assists in optimizing patient care, facilitating personalized treatment approaches, and improving overall patient outcomes.

However, there are concerns regarding the accuracy of cytological diagnosis, which vary depending on the category. The accuracy is higher for negative and malignant categories, at 76% and 81%, respectively [8]. On the other hand, for the intermediate suspicious malignancy cases, there is a lower agreement observed, with rates of 32% and 22%, respectively. In the previous study, the overall agreement among pathologists for cytological diagnosis was 68% [8]. A recent meta-analysis reported that overall diagnostic sensitivity for malignant pleural effusions was 58% [9,10]. Cytological diagnosis varies according to the type of malignancy, particularly in primary thoracic malignancies. The sensitivity for lung adenocarcinoma was found to be 83.6%, while for lung squamous cell carcinoma, it was 24.2% [9,10]. The report revealed that the diagnostic sensitivity of malignant pleural effusion for breast cancer was 65.3%, indicating a relatively low performance as a screening tool. Suspected malignant pleural effusion patients with negative cytology may need flow cytometry, tissue biopsy, and other tests for evaluation. Flow cytometry is useful for malignant hematological effusions, while immunochemistry and fluorescence in situ hybridization aid in identifying non-hematologic malignancies [11]. However, these tests can be costly and time-consuming. The limitations of cytological diagnosis, including its subjective nature and potential for human error, have driven researchers to explore alternative approaches that offer more reliable and accurate assessments. Consequently, researchers have actively utilized deep learning methods to diagnose cytological images and address the drawbacks and weaknesses associated with conventional cytology approaches. This study aimed to assess the diagnostic accuracy of AI in detecting malignant pleural effusion cytology, specifically that related to breast cancer. This will be accomplished by analyzing a larger number of cytopathology slides gathered from multiple institutions across the nation. Additionally, this study aimed to utilize z-stacked cytology slide images to develop an AI model.

Deep learning-based artificial intelligence (AI) image analysis technology has recently been actively researched in the medical field, and it has been showing remarkable results [12,13,14]. In addition, a recently published study on the classification of lung cancer cells in pleural fluid using a deep convolutional neural network showed promising results [10]. Several studies have already indicated that AI achieves diagnostic accuracy equal to or better than that of human experts in the diagnosis of cervical cytology slide images [7,8,9]. AI models have been increasingly used in cytology, with an initial focus on the examination of gynecological samples, showing promising results [15]. Currently, AI models are used to classify various nongynecological samples, including urinary tract cytology, thyroid fine needle aspiration cytology (FNAC), breast FNAC, and pleural fluids [16,17,18,19]. However, only a few studies have been conducted on AI classification in malignant serous fluid cytology [12,19]. In addition, no studies have diagnosed breast cancer in pleural fluid using AI to the best of our knowledge, and only two studies on breast cancer FNAC using artificial neural networks have been published [19,20]. These studies have shown perfect results in distinguishing benign from malignant tumors. However, the number of cases was relatively small; therefore, it was necessary to use a larger number of slide images for validation. Furthermore, many studies have employed image datasets to develop AI models for cytological diagnosis; however, these datasets often lack z-stacking images. For this study, we collected a larger number of cytopathology slides with z-stacked images and developed an AI model.

## 2. Materials and Methods

**Figure 1:**
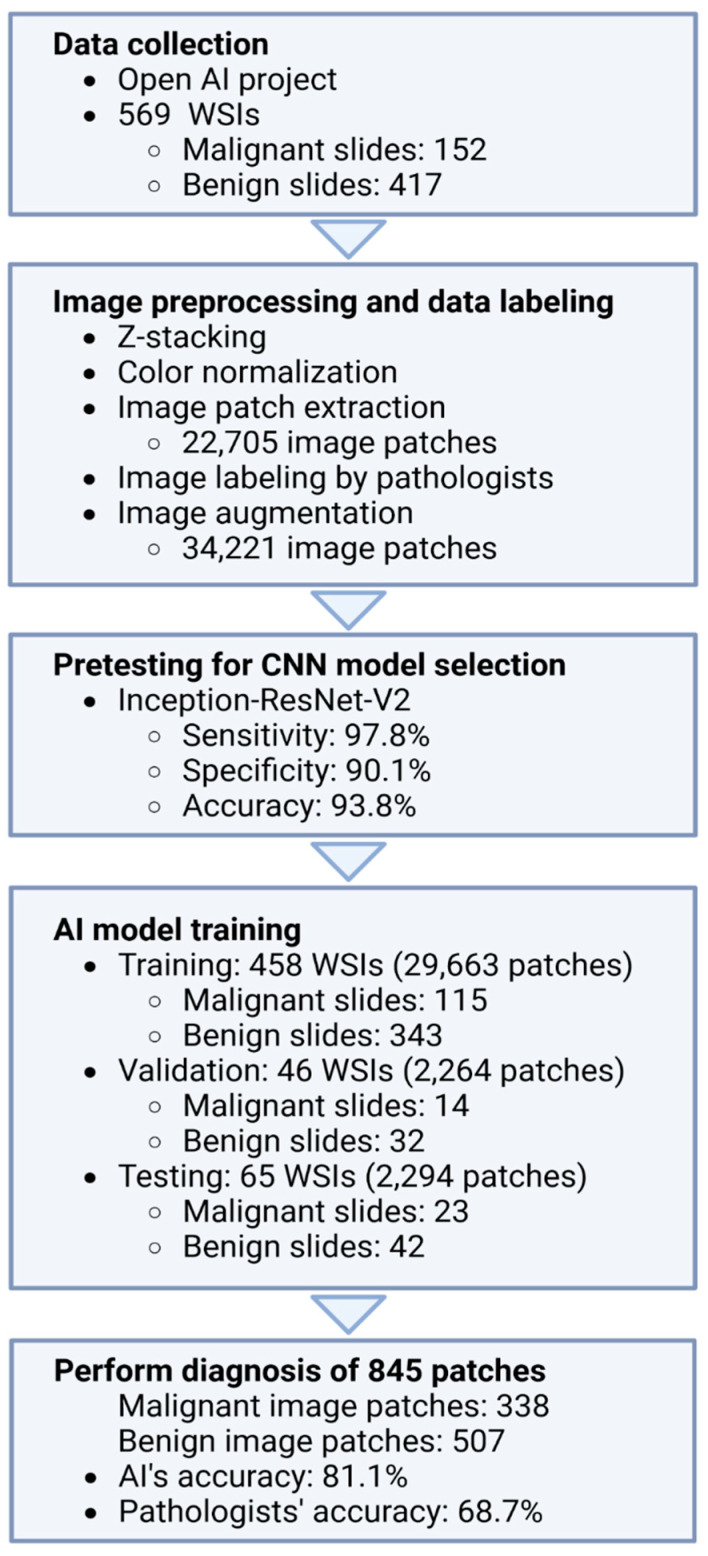
Overview of the study.

This study was approved by the Institutional Review Boards of the Catholic University of Korea, College of Medicine (UC21SNSI0064); Yonsei University College of Medicine (4-2021-0569); the National Cancer Center (NCC2021-0145); and St. Vincent Hospital, Catholic University of Korea, College of Medicine (VC22RISI0131). An outline of the method for classifying malignant and benign cells in pleural cell slide images of breast cancer patients using a deep convolutional neural network model (DCNN) is presented in Figure 1.

### 2.1. Data Collection

In this study, we used 569 cytologic whole-slide images (WSIs) collected from the Open AI Dataset project for cytopathology in 2021 (https://www.aihub.or.kr accessed on 19 June 2023). In collaboration with the Korean Society of Cytopathology, the Catholic University of Korea Uijeongbu St. Mary’s Hospital, the National Cancer Center, the Korea Cancer Center Hospital, and ten other medical institutions constructed a digital cytology learning dataset. This dataset was created by refining, labeling, storing, and quality-controlling cytopathology images, consisting of 5506 WSIs for ten types of cancer and benign cases. Furthermore, 207,037 patch images were extracted from this dataset. All malignant cytopathological slides were confirmed via histological examination. The cytopathological dataset collection and preparation processes are shown in Supplementary Figures S1 and S2, respectively.

### 2.2. Image Preprocessing 

**Figure 2:**
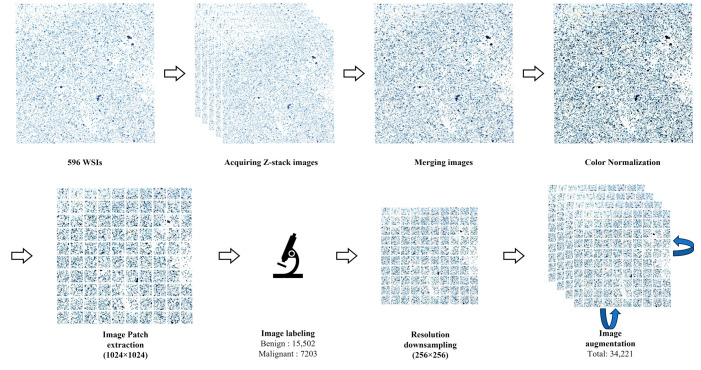
Overview of image preparation in the study. All cytology slides were scanned in multiple layers by z-stacking and merged to obtain an image with appropriate focus. WSIs were segmented into small image patches and labeled by pathologists as benign or malignant. Image augmentation was performed after labeling.

The collected WSIs were scanned as extended depth-of-field images by merging multilayered z-stacked images through slide scanners to correct for image defocusing. The scanners used were AT2 (Leica Biosystems, Nussloch, Germany), Pannoramic Flash 250 III (3DHISTECH, Budapest, Hungary), and NanoZoomer S360 (Hamamatsu, Shizuoka, Japan). After obtaining the WSIs, color normalization was performed for color constancy. Then, we split those images into non-overlapping small image patches of 1024 × 1024 pixels. Pathologists classified and labeled the split images as malignant or benign. After labeling, downsampling was performed to convert the images into 256 × 256-pixel images. Finally, the image patch data were augmented via horizontal flip, vertical flip, and clockwise rotation to improve the sufficiency and diversity of the training data (Figure 2).

### 2.3. Pretesting for DCNN Model Selection

We pretested several DCNN models, including Inception ResNet-V2, Efficientnet-b1, ResNext50, Mobilenet v2, Densenet 121, and ResNet 50. A total of 344 WSIs, including 152 malignant and 192 benign, were used in the pre-testing. After preprocessing, 14,526 image patches were acquired (7203 malignant/7323 benign). We used 11,575 patch images (5765 malignant/5810 benign) for training, 1475 patch images (722 malignant/756 benign) for validation, and 1473 patch images (716 malignant/757 benign) for testing. After a comparison of the performances, we selected Inception-ResNet-V2 as the model for this study. To prevent overfitting, we employed data augmentation techniques. Additionally, we implemented early stopping to obtain the best possible model. The data were made more quantitative by extracting patches from WSIs.

### 2.4. AI Model Training

**Figure 3:**
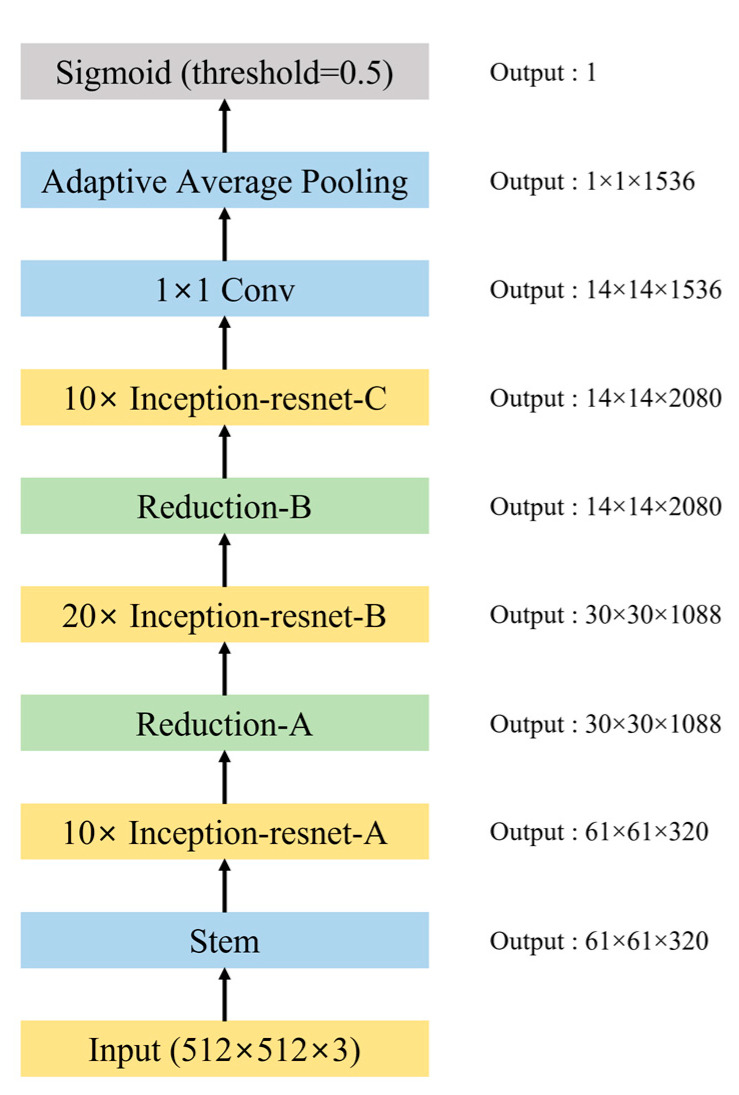
Schema of Inception-ResNet-V2 network used in this study for binary classification.

We experimented with 165-layer Inception-ResNet-v2, as in Figure 3. The Inception-Resnet-V2 model is used in this experiment. Inception-ResNet-V2 is a convolutional neural network developed by Google that has satisfactory performance in image analysis. The Inception-ResNet approach combines the residual network with an inception architecture previously developed by Google [21]. A residual network is a neural network designed to address the issue of increasing training and test errors as the number of layers significantly increases in existing deep learning. In addition, residual networks use shortcut connections to jump directly to the classification stage when training reaches the optimal depth, thus enabling faster training than Inception V3 [21,22]. In this experiment, we substituted the softmax function in the output layer with a sigmoid function for the binary classification. The augmented patch images were randomly assigned to train, validate, and test the DCNN model (Table 1). The malignant probability of each image patch was estimated; if the probability was 50% or more, the image patch was classified as malignant. The dataset was divided into three parts, namely training, validation, and testing, following an 8:1:1 ratio as specified. The patch-wise distribution of positive and negative images is well balanced, with a ratio of 6:5. Details of the number of WSIs/image patches used for the DCNN model are shown in Table 1.

### 2.5. Comparison of the Performance of the AI Model and the Pathologists

To evaluate the performance of the AI model, we randomly selected 845 Pap smear images, including 338 malignant and 507 benign, from the 34,221 image patches. Subsequently, three pathologists and the AI model diagnosed the patch images, and the results were compared. After comparison, each pathologist re-diagnosed the patch images that were inconsistent with the AI diagnosis.

## 3. Results

### 3.1. Data Characteristics

We collected Papanicolaou-stained 569 WSIs, consisting of 417 benign and 152 metastatic breast carcinoma cases, which included 564 conventional smears and 5 liquid-based prep (LBP) slides. The WSIs contained at least three z-stack layers, and 94.9% of the WSIs were scanned using a Pannoramic Flash 250 III (3DHISTECH, Budapest, Hungary). Table 2 shows the characteristics of the cytological slides used in this study.

### 3.2. Pretesting for DCNN Model Selection

The overall accuracy of the image classification ranged from 89.48% to 93.82%. Inception ResNet v2 showed the highest accuracy of 93.8%, whereas Efficientnet-b1, ResNext50, MobileNet v2, Densenet 121, and ResNet 50 showed accuracies of 92.7, 93.5, 89.5, 92.5, and 91.9%, respectively. Among the DCNN models, Inception-ResNet-v2 showed the most accurate results, with a sensitivity and specificity of 97.77 and 90.09%, respectively. The comparison of the performances of the models with the pre-testing model selection results is listed in Table 3.

### 3.3. AI Model Training

The Inception ResNet v2 model showed accuracy, sensitivity, and specificity values of 95.0, 93.4, and 95.8%, respectively, for an oversampled training dataset. The model showed accuracy, sensitivity, and specificity values of 92.9, 87.6, and 96.6%, respectively, when using all training datasets and 93.8, 97.8, and 90.1%, respectively, when using a reduced training dataset.

### 3.4. Comparison of the Performances of the AI Model and Pathologists

The average of the three pathologists showed 72.49% sensitivity, 88.89% specificity, and 68.74% accuracy with a Fleiss kappa coefficient of 0.482. Pathologist A showed a sensitivity of 67.8%, a specificity of 94.9%, and an accuracy of 70.2%; Pathologist B showed a sensitivity of 56.8%, a specificity of 98.0%, and an accuracy of 68.1%; and Pathologist C showed a sensitivity of 92.9%, a specificity of 73.8%, and an accuracy of 68.0%. The AI showed a sensitivity of 95.0%, a specificity of 98.6%, and an accuracy of 81.1%.

**Figure 4:**
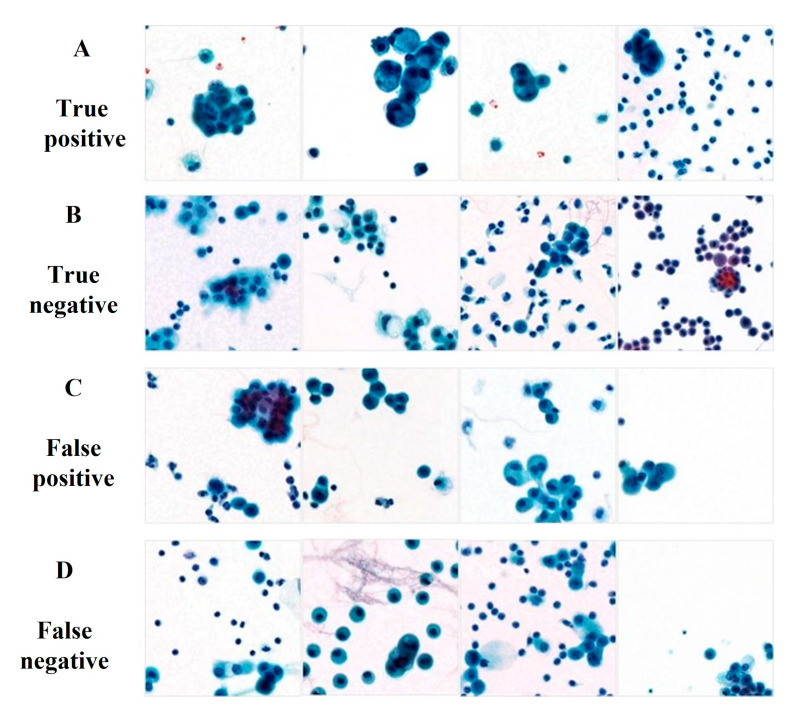
Images classified by AI. Patch images accurately classified patch images by the AI: (**A**) Malignant and (**B**) benign. Patch images misdiagnosed by AI (**C**) benign cells as malignant, and (**D**) malignant cells as benign.

**Figure 5:**
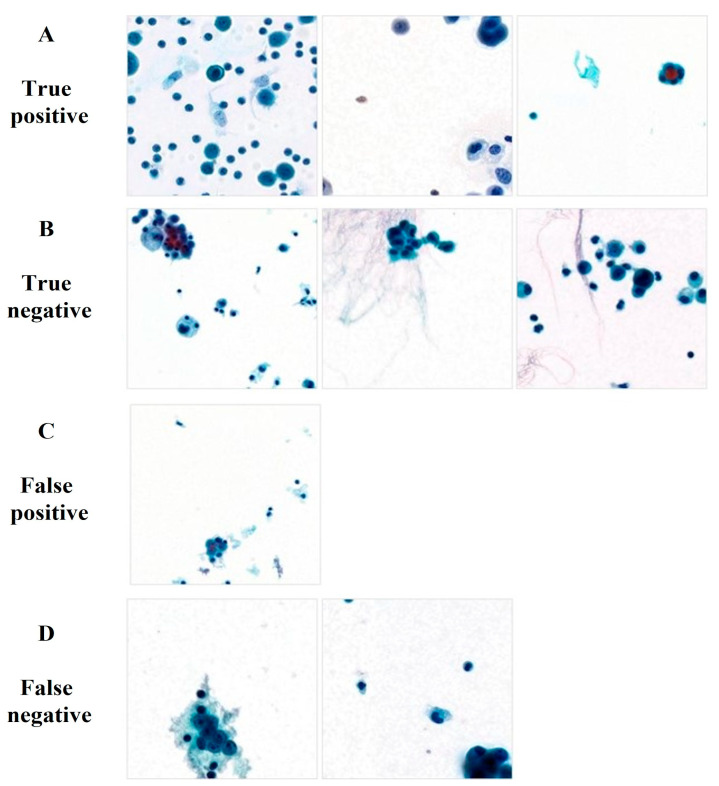
Patch images correctly or incorrectly diagnosed by AI. Patch images the correctly diagnosed by the AI but misdiagnosed by pathologists: (**A**) Malignant and (**B**) benign. Patch images correctly diagnosed by pathologists but misdiagnosed by the AI: (**C**) Benign and (**D**) malignant.

The AI diagnosed 321 out of 845 true positives, 500 true negatives, 7 false positives, and 17 false negatives (Table 4). Examples of AI-diagnosed images are shown in Figure 4. In cases where both the AI and a pathologist made diagnoses, the AI alone correctly diagnosed 18 true positives and 3 true negatives. There was one false positive and two false negatives when only the AI was incorrect. Images showing the discrepancies in the diagnosis by the AI and pathologists are shown in Figure 5.

**Figure 6:**
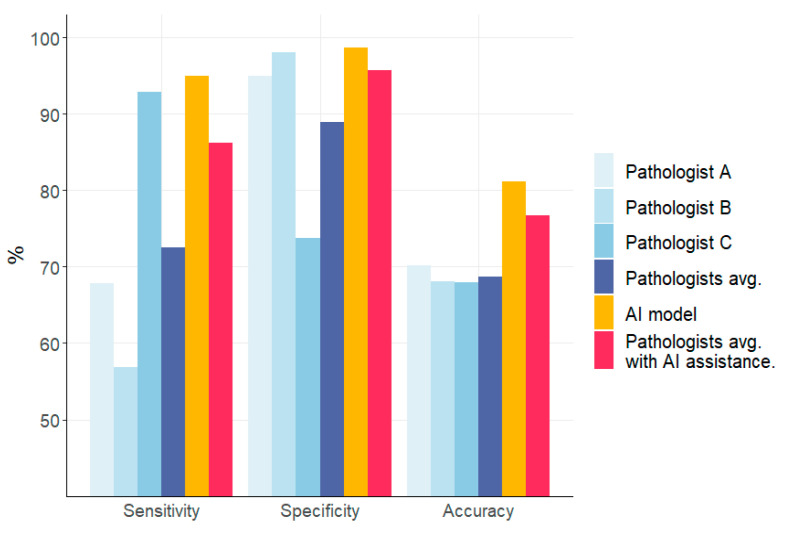
Comparison of the performance of the AI model and human pathologists.

Cases with inconsistencies in the diagnosis by the AI and the pathologist were re-examined by all pathologists, and the average result improved to 86.19% sensitivity, 95.66% specificity, and 76.71% accuracy (Figure 6). In addition, the kappa coefficient for the pathologists improved to 0.806. The AI diagnosed 7 false-negative and 17 false-positive cases.

## 4. Discussion

Diagnosing pathological WSIs in various organs using AI is currently showing considerable progress, with various patents and applications in the medical field [23,24]. A recent systematic review found that there are not many studies that have used artificial neural networks in the field of effusion cytology [25]. Consequently, additional research is imperative for exploring the potential of neural networks to aid in diagnostic cytology. In this study, we successfully demonstrated that AI exhibited remarkable accuracy in diagnosing breast cancer pleural effusion cytopathology for the first time. Our research is the first of its kind to use the largest dataset that includes z-stacking.

In this study, we used 569 qualitatively assured WSIs obtained from various medical institutions nationwide. The sample size was sufficiently large and exhibited a high level of demographic diversity and heterogeneity, surpassing the findings of previous studies. Moreover, we employed merged z-stacked images in our study, which resulted in an improved focus. The AI model developed for this experiment exhibited an accuracy of 81.13% in classifying patch images, outperforming the average accuracy of 72.49% achieved by experienced pathologists. Furthermore, when the cases in which the AI and pathologists disagreed on the diagnosis were reevaluated, the pathologists’ diagnostic accuracy improved. This suggests that AI assistance can enhance cytopathologists’ interpretive capabilities by striking a balance between sensitivity and specificity.

The morphological features of metastatic breast carcinomas are variable and can exhibit non-cohesive isolated cells, large cell balls, and linear arrangements [26,27]. Diagnosing metastatic breast carcinoma in pleural fluid is challenging even for experienced pathologists, especially when dealing with predominantly isolated cell patterns that are difficult to differentiate from reactive mesothelial cells and histiocytes [28]. Pleural fluid can serve as a culture medium for floating cells, leading to a decrease in the nuclear-to-cytoplasmic ratio compared to typical cancer cells; this phenomenon can mimic the appearance of mimicking normal or reactive cells [29]. Mesothelial cells can be readily activated and mimic phenotypic traits reminiscent of malignant cells, particularly when exposed to inflammatory stimuli such as pneumonia or tuberculosis. Macrophages, which are integral components of the mononuclear phagocytic system, can be activated in response to pleural effusion or inflammation. This activation can result in macrophages exhibiting characteristics that resemble malignant cells. Breast cancer cells, particularly metastatic lobular carcinomas, possess distinct characteristics such as smaller size and a more blended appearance that differentiate them from other types of carcinomas such as lung cancer [30,31,32,33,34]. This disparity in cellular morphology poses a challenge for accurately diagnosing these conditions via pleural fluid cytology.

In this study, AI performed well in identifying and classifying cells with distinct malignant features, such as nuclear hyperchromasia, pleomorphism, a high N:C ratio, and nuclear overlapping (Figure 4A). In addition, AI helped diagnose a small number of atypical cells that are difficult for humans to recognize (Figure 5A). However, it also tended to misdiagnose as malignant if the mesothelial cells showed severe pleomorphism, hyperchromasia, or nuclear overlap (Figure 4C and Figure 5). In addition, there were cases in which AI could not correctly classify malignant cells because of an unclear cytoplasmic border, a low N:C ratio, or other unknown reasons (Figure 4D and Figure 5C).

Inception-ResNet-V2 was introduced in 2016 and showed satisfactory top-1 and top-5 accuracies of 77.8 and 94.1%, respectively, in the ImageNet examination [35]. In addition, Inception-ResNet-V2 has been used in many pathological image and medical data diagnostic studies and has shown promising results. For example, a study in which Inception-ResNet-V2 classified tissue slides of skin melanoma reported high accuracy (96.5%), sensitivity (95.7%), and specificity (97.7%) [36]. Moreover, a study published in 2022, which analyzed cervical Pap smear cytology images, reported a compliance accuracy of 96.44% [37].

Currently, numerous AI-based models are emerging to aid pathologists in the diagnosis of breast cancer. Many AI-based applications have been developed in the field of breast pathology, including primary tumor detection, breast cancer grading, the identification of histological subtypes of breast cancer, analysis of mitotic figures, and the prediction of survival outcomes [38,39,40,41,42]. Recently, AI algorithms have been developed to offer quantitative analysis of immunohistochemistry-stained images, specifically for evaluating the KI-67, estrogen receptor (ER), progesterone receptor (PR), and human epidermal growth factor receptor-2 (HER2) markers [41,43,44,45]. A recent study successfully developed an AI model for diagnosing lymph node metastasis in breast cancer, achieving an impressive accuracy of 98.8% [46]. Another study developed an AI model using pleural effusion cell blocks from breast, gastric, and lung carcinoma [47]. This model achieved an area under the curve (AUC) of 0.91, identifying benign and malignant pleural effusion, and successfully determined the primary site of metastatic carcinoma [47]. Previously, an AI model was developed to diagnose metastatic carcinoma using effusion cytology. The dataset used in this study consisted of 57 benign cases, 54 metastatic adenocarcinoma cases, 1 squamous cell carcinoma case, and 1 signet ring carcinoma case [48]. Although this study achieved an impressive 100% accuracy, the dataset used was relatively small [48]. This study did not clarify the number of patients with metastatic breast cancer pleural effusion that were included in the analysis. Our present study differs from previous studies in that this AI model specifically targeted breast cancer metastasis using a larger dataset. In another study, a computer-aided diagnostic approach was employed utilizing the nuclear structure of mesothelial cells to classify malignant mesothelioma in effusion cytology specimens [49]. This study also achieved 100% accuracy; however, it was based on a small dataset that included 16 cases of malignant mesothelioma and 18 cases of benign pleural effusion [49]. Recently, a few studies have employed AI for the FNAC cytology of breast cancer. Dey et al. successfully identified lobular carcinomas in FNAC samples [19]. Another study used an AI model to accurately identify fibroadenomas and infiltrating carcinomas of the breast in FNAC cases with 100% sensitivity and specificity [20]. However, these studies used small datasets. In this study, we utilized a larger dataset comprising 596 cytological WSIs of metastatic breast cancer, collected from various universities and hospitals throughout South Korea. Typically, malignant pleural effusion fluids contain a high number of benign background cells, leading to a low population of tumor cells. False-negative or inconclusive results may occur in samples with a low number of cancer cells during morphological analysis. The findings of the current study demonstrated superior accuracy, sensitivity, and specificity compared with pathologists in diagnosing malignant pleural effusion cytology related to breast cancer.

This study has some limitations. First, the diagnosis was limited to patch images and was not applied to the diagnosis of WSIs. Therefore, it is necessary to construct the malignancy probability of each image tile and set a cutoff value for the probability of all tiles. In addition, we divided the classification into malignant and benign two-tiers and did not designate the atypical category. Therefore, if a few atypical cells are present on WSI, this model will likely lead to an inappropriate diagnosis. Second, this AI model does not explain why cells are viewed as malignant or benign, which can confuse the pathologist and lead to an inappropriate diagnosis when the diagnoses of the pathologist and the AI do not match. Due to the black-box nature of AI systems, the mechanism by which they arrive at conclusions is not well understood. Therefore, it is necessary to introduce explainable AI (XAI) to address this shortcoming [50,51]. XAI focuses on developing AI models that can be understood by humans. XAI is used to create AI models that can not only make accurate predictions but also provide an explanation of how they arrived at those predictions. When the diagnoses of the pathologist and AI do not match, the pathologist can make a diagnosis more accurately and quickly by receiving real-time feedback from AI [52].

In future studies, the application of this AI model to WSI diagnosis and further validation with external WSIs will be necessary. If this model undergoes validation using data from a multi-ethnic population, then the performance of the model can be generalized. In addition, it is necessary to develop a more accurate AI model. Currently, various AI image classification models, showing higher classification accuracy and potential for use in pathological image diagnosis, are being actively studied. Xie et al. reported that the noisy student training (EfficientNet-L2) method, which comprised pseudo-labeled images in the training dataset to add noise inside the dataset, improved the top-1 and top-5 accuracies of ImageNet classification to 88.4 and 98.7%, respectively [53]. In 2021, Pham et al. announced meta-pseudo labels and semi-supervised learning that improved noisy student training and enhanced the top-1 and top-5 accuracies of ImageNet classification to 90.2 and 98.8%, respectively [54].

The second aspect to be investigated is the use of fewer computer resources for model training to increase availability. In general, DCNN training requires increased computing power and data storage as accuracy increases. MobileNet is a lightweight convolutional neural network architecture for mobile and embedded vision applications. MobileNet uses depthwise separable convolutions to reduce the number of parameters and computations required for image classification. The top-1 and top-5 accuracies of this model on the ImageNet dataset are above 70 and 89%, respectively [55]. In addition, EfficientNet uses fewer parameters and calculations and is a relatively accurate model. EfficientNet-B0 showed top-1 and top-5 accuracies of 77.1 and 93.3%, respectively, in the ImageNet tests. In addition, EfficientNet-B7 showed top-1 and top-5 accuracies of 84.3 and 97.0%, respectively, but required more computer resources [56]. Other DCNN techniques can be considered when lower resources and faster training rates are required.

## 5. Conclusions

The AI model developed for this experiment exhibited an accuracy of 81.13% in classifying patch images, outperforming the average accuracy of 72.49% achieved by experienced pathologists. Cases with inconsistencies in the diagnosis by the AI and the pathologist were re-examined and the average accuracy, sensitivity, and specificity of the pathologists improved to 87.9, 80.2, and 95.7%, respectively. This study demonstrated that AI could provide significant accuracy in diagnosing breast cancer pleural effusion cytopathology, which could enhance the interpretative ability of cytopathologists. Future studies should focus on applying this AI model to WSI diagnosis, validating it with external WSIs. Additionally, efforts should be directed towards exploring methods to develop a more accurate AI model that requires fewer computer resources for model training, thereby increasing its availability.

## Figures and Tables

**Table 1:** Number of whole-slide images used for the deep convoluted neural network (DCNN) model.

	No. of Whole Slide Images (Image Patches)			
	Training	Validation	Testing	Total
Benign	330 (12,389)	44 (1545)	43 (1568)	417 (15,502)
Malignant	111 (17,274)	18 (719)	23 (726)	152 (18,179)
Total	441 (29,663)	62 (2264)	66 (2294)	569 (34,221)

**Table 2:** Data characteristics of the enrolled cytologic slides.

Characteristics	Number of Cases (*n* = 569)
Age (median)	18–104 (66)
Cytologic diagnosis	
Malignant lesions	152 (26.7%)
Benign lesions	417 (73.3%)
Preparation method	
Conventional	564 (99.1%)
Liquid-based preparation	5 (0.9%)
Z-stack layers	
3-layers	490 (86.1%)
5-layers	79 (13.9%)
Scanner	
Pannoramic Flash 250 III (3DHISTECH)	540 (94.9%)
AT2 (Leica)	2 (0.4%)
NanoZoomer S360 (Hamamatsu)	27 (4.7%)

**Table 3:** Pretesting performance of DCNN models. Inception-ResNet-V2 exhibited the highest accuracy and best performance among the pretesting models.

	Accuracy	Sensitivity	Specificity
Inception ResNet v2	0.9382	0.9777	0.9009
Efficientnet-b1	0.9267	0.8855	0.9657
ResNext50	0.9348	0.9679	0.9036
Mobilenet v2	0.8948	0.9162	0.8745
Densenet 121	0.9246	0.9218	0.9273
ResNet 50	0.9192	0.8980	0.9392

**Table 4:** Confusion matrix of the AI classification.

Actual Diagnosis	AI Diagnosis	
Total (*n* = 845)	Malignant (328)	Benign (517)
Malignant (338)	True positive (321)	False negative (17)
Benign (507)	False positive (7)	True negative (500)

## Data Availability

The data presented in this study are available upon request from the corresponding author.

## Supplementary Materials

The following supporting information can be downloaded at: https://www.mdpi.com/article/10.3390/cells12141847/s1, Figure S1: 2021 NIA project for cytopathology dataset; Figure S2: Dataset preparation process.
