# Supplementary material for: Deep Learning-Based Computational Cytopathologic Diagnosis of Metastatic Breast Carcinoma in Pleural Fluid
Source: Cells. 2023 Jul 13;12(14):1847. doi: 10.3390/cells12141847 (PMC10377793; doi:10.3390/cells12141847)
Supplement: Supplementary file 1 [file cells-12-01847-s001.zip › cells-2486185-supplementary.pdf]

Supplementary Figures:

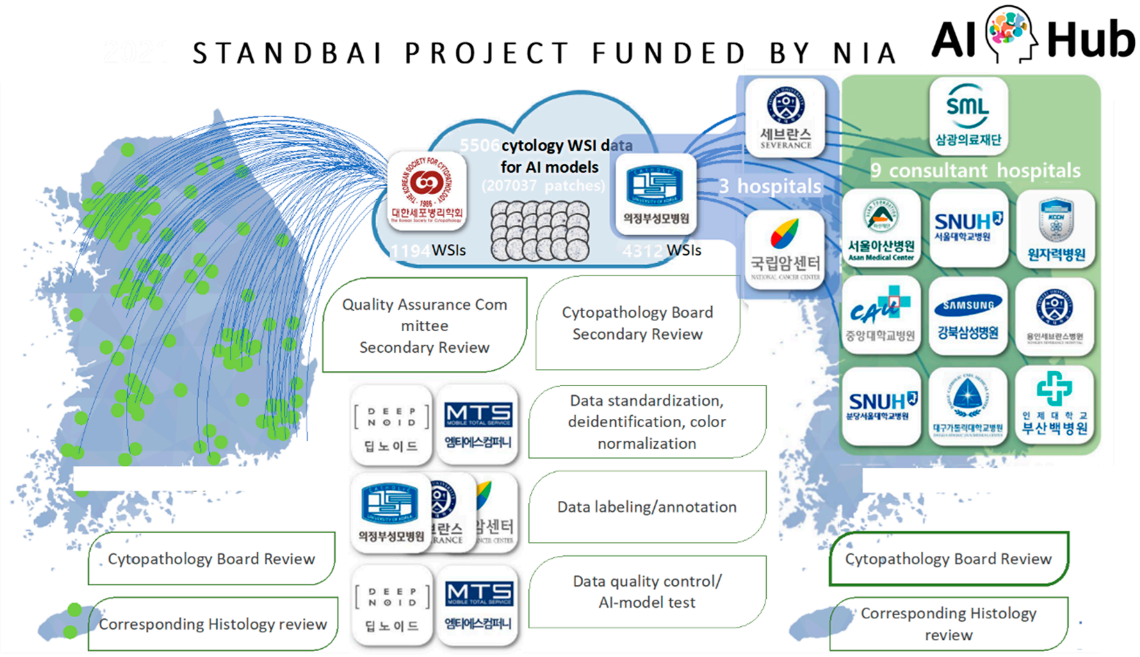

Figure S1. 2021 NIA project for cytopathology dataset.

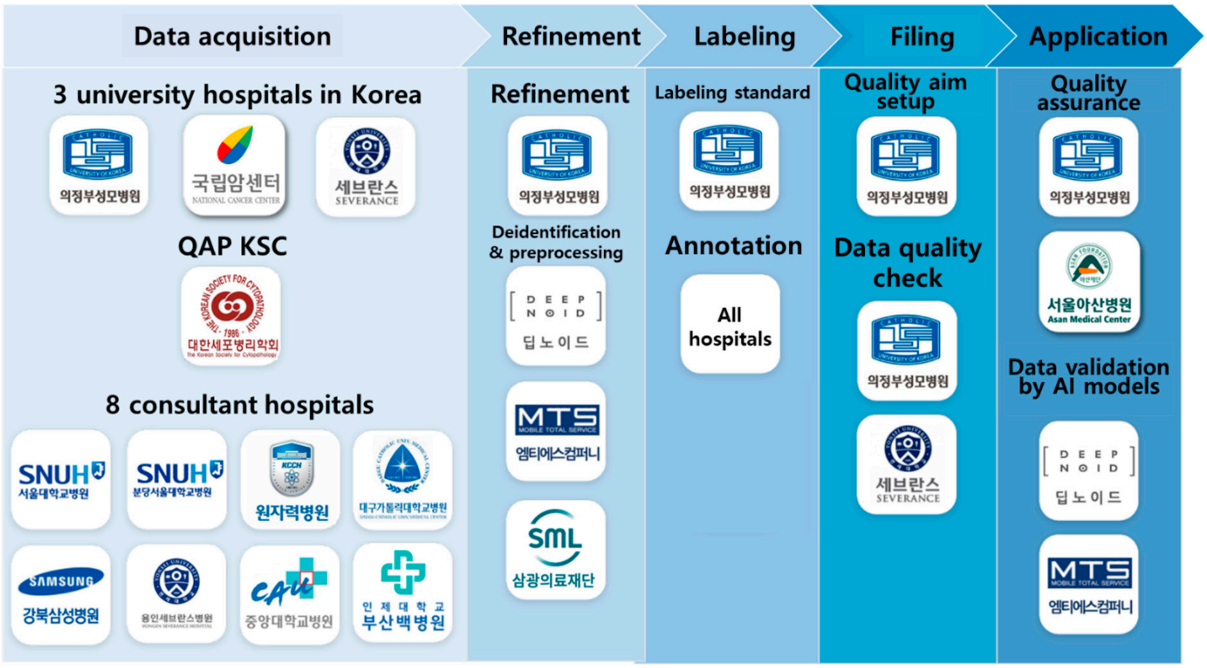

Figure S2. Dataset preparation process.
